# Fatty Acid Profile, Phytochemicals, and Other Substances in *Canarium odontophyllum* Fat Extracted Using Supercritical Carbon Dioxide

**DOI:** 10.3389/fchem.2019.00005

**Published:** 2019-01-31

**Authors:** Hock Eng Khoo, Azrina Azlan, Noor Atiqah Aizan Abd Kadir

**Affiliations:** ^1^Department of Nutrition and Dietetics, Faculty of Medicine and Health Sciences, Universiti Putra Malaysia, UPM Serdang, Malaysia; ^2^Research Centre of Excellence for Nutrition and Non-Communicable Diseases, Faculty of Medicine and Health Sciences, Universiti Putra Malaysia, UPM Serdang, Malaysia

**Keywords:** dabai, LCMS, oil, oleoresin, peptide, terpenoid, volatile

## Abstract

This study aims to identify potential phenolic compounds, terpenoids, and other phytochemicals, as well as fatty acid profile and peptides in *Canarium odontophyllum* (CO) oil and oleoresin, extracted using supercritical carbon dioxide. LC-ESI-MS was applied in separation and tentative identification of phytochemicals in CO oil and oleoresin. Based on the results, 11 common fatty acids and their isomers, monoglycerides, diglycerides, as well as other types of lipid, were tentatively identified in the CO oil and oleoresin. The identified fatty acids consisted of saturated fatty acids (C8–C16), monounsaturated fatty acids (C16:1 and C18:1), polyunsaturated fatty acids (C18:2, C18:3, C18:4, and C20:3), and other unclassified fatty acids. The tentatively identified phenolic compounds were phenolic acids, flavonoids, lignans, and a phenolic monoester. Triterpenes, sesquiterpenes, and apocarotenoids were the terpenoids found in CO oil and oleoresin. Besides these typical bioactives, some volatiles, aromatic compounds, peptides, and other known and unknown phytochemicals were also tentatively identified in the oil and oleoresin of CO. Some of these compounds are new compounds identified in CO oil and oleoresin, which are not found in many other fruit oils. Although CO oil and oleoresin contain a small number of phytochemicals, their contribution as antioxidants may prevent several diseases. In this study, we hypothesized that CO oleoresin contains certain types of fatty acids that render its semi-solid together with other chemical components which are not found in CO oil. This is the first study that tentatively identified fatty acids, peptides, and potential phytochemicals in CO oil and oleoresin using LC-ESI-MS.

## Introduction

The *Canarium odontophyllum* Miq. fruit, also known as dabai, is one of the species of *Canarium* fruits from the Burseraceae family that exists in the Southeast Asian region. The trees of *Canarium odontophyllum* (CO) are largely cultivated in the Borneo region, such as the states of Sarawak and Sabah. Physical description of dabai has been reported previously; the fruit is usually dark purple to black in color, and it has oily pulp. CO fruit is 3.7–4.1 cm in length and 2.4–2.8 cm in width, with a hard 3-angular-seed (Khoo, 2014).

Nutritional values and antioxidant properties of the CO fruit have been reported in our previous studies. The CO pulp has a high oil content (Chew et al., 2011). The fat content of CO fruit ranged between 21.16 ± 4.71 and 25.76 ± 3.03 g/100 g fresh weight. According to Azlan et al. (2010), the CO pulp contained 44.4% total saturated fats, 42.8% total monounsaturated fats, and 12.8% total polyunsaturated fats. Besides total fatty acids, the study also reported that the CO oil had, as the main fatty acids, 36.1% palmitic acid, 5.8% myristic acid, 41.5% oleic acid, and 11.8% linoleic acid. In addition to the balanced fatty acid composition, CO pulp had moisture, total available carbohydrate, protein, and ash content of 50.4–51.9%, 4.5–9.2%, 3.5–5.2%, and 1.7–1.9%, respectively; mineral content, amino acid composition, as well as antioxidative properties, were also determined in the CO pulp (Chew et al., 2011).

The extracts of defatted CO pulp have been shown to possess antioxidative properties and cardioprotective effects (Khoo et al., 2014). The defatted CO pulp contained anthocyanins as the major antioxidants. The anthocyanin-rich defatted CO pulp significantly reduced plasma total cholesterol and plasma LDL-cholesterol of the hypercholesterolemic rabbits compared to the positive control (Shakirin et al., 2012b). Aqueous extract of CO leaves was also able to reduce high blood pressure through vasodilation of the rat thoracic aorta (Basri et al., 2018).

The balanced fatty acid composition in CO oil demonstrated a protective effect in healthy rabbits (Shakirin et al., 2012a). In the study, Shakirin et al. (2012a) reported that CO oil brought about a significant improvement in lipid profile (increased plasma HDL-cholesterol, and reduced plasma LDL-cholesterol and triglyceride levels), as well as reduced TBARS (thiobarbituric acid reactive substances) level and favorable changes in the levels of plasma malonaldehyde, superoxide dismutase, glutathione peroxidase, and total antioxidant status. The oil had a total phenolic content of 20.27 mg gallic acid equivalent/100 g oil. The high amount of phenolic compounds determined in CO pulp oil could contribute to the protective effect of the fruit *in vivo*.

Extraction method and type of solvent used are essential for extracting nutrients and phytochemicals from a high-fat fruit. As dabai is a high-fat fruit, the fruit pulp contains both polar and non-polar phytochemicals. Although methanol has been recognized as the widely used solvent for extraction and isolation of chemical components from plants, it is known to be toxic to the human body if ingested in even a small amount. The use of greener solvents such as water and carbon dioxide is recommended. However, water is a polar solvent, and it is hydrophilic. For that reason, supercritical carbon dioxide (SCO_2_) is the best to be used for extracting nutrients and phytochemicals with a wide range of polarity from any sources (Mukhopadhyay, 2009). CO_2_ is also non-toxic and recyclable. The solubility behavior of the non-polar phytochemicals in SCO_2_ has been reported in a previous study (Güçlü-Üstündag and Temelli, 2004). The study reported that the solubility of the compounds is dependent on the operating conditions. The use of an optimized extracting condition warrants optimal extraction yield with more types of chemical components isolated from a plant sample.

So far, except for the proximate analyzes and antioxidant properties and the determination of fatty acid and amino acid profile, as well as selected vitamins and minerals, limited studies have been done on the chemical analysis of CO oil. There has been no report on the chemical properties of SCO_2_ extracted CO oil and oleoresin. Due to the lack of data for the complete identification of phytochemicals or plant metabolites and nutritional components in SCO_2_ extracted CO oil and oleoresin, there is a need to characterize and to identify potential chemical components in the fat samples. This study was also performed to identify fatty acids, peptides, and potential phytochemicals in CO oil and oleoresin using LC-ESI-MS technique, as well as to quantify the fatty acid composition in the oil and oleoresin. Moreover, this is the first study on the qualitative analysis of chemical components in CO oil and oleoresin.

## Materials and Methods

### Chemicals and Reagents

Acetonitrile of LiChrosolv grade and methanol used in the fractionation of phytochemicals, peptides, and fatty acids were purchased from Merck (Darmstadt, Germany). Water was purified by a Milli-Q system from Millipore (Milford, USA).

### Preparation of Sample and Extraction

The CO fruit (200 kg) was purchased from an identified supplier through Semongok Agriculture Research Centre, Sarawak. About 100 kg of blanched CO pulp (at 60°C for 15 min) was obtained upon removal of the kernel. The CO pulp was freeze-dried using a benchtop freeze-drier (Virtis, NY, USA), and the lyophilized pulp was pulverized using a household blender (Panasonic, Malaysia) and sieved by a 20-mesh plastic sieve. Lipids from the lyophilized samples were extracted using an ABRP200 SCO_2_ extractor (Thar Process, PA, USA).

We have previously optimized the SCO_2_ extraction conditions and established a laboratory protocol for the CO fat extraction (Jelani et al., 2017). However, the extraction of CO fat was done based on the semi-pilot scale, and the optimized extraction condition was applied. In this study, CO fat was extracted from the CO pulp powder using the SCO_2_ extractor. The extraction was done by the automated system with the heater set to 40°C and with an extraction pressure of 40 MPa.

The extraction of CO oil was done using the extractor with a volume of 500 ml. Before the injection of CO_2_ into the extraction vessel, 50 kg of the lyophilized sample was inserted into the vessel. The extraction was done based on the optimized conditions for CO oil extraction as follows: an extraction pressure of 40 MPa and an extraction temperature of 40°C. The flow of SCO_2_ was maintained at 15 g/min, and the static duration was set to 30 min. The CO pulp powder was soaked in the vessel containing the SCO_2_ at high pressure for 30 min. After 30 min of soaking, the oleoresin and oil were removed from the separation vessel via a manual valve after the extraction was completed. The extraction process took a total of 2 h. The extracted oil was collected as CO oil, and the semi-solid fraction was collected as CO oleoresin. Extraction of oil was performed in three replicates.

Semi-polar extracts of the collected oil and oleoresin were obtained by extraction with aqueous methanol. The extraction was previously optimized using several extraction solvents, although methanol was found to be the ideal extraction solvent for extracting phytochemicals from CO oil, especially polyphenols, terpenoids, and glycosides (Biswas et al., 2013). The chemical components of CO oil and oleoresin were extracted using aqueous 95% methanol (1:10 *w/v*) at room temperature for 20 min. The oil layer was separated using a separating funnel. The methanolic extracts were filtered using 0.45 nm Sartorius syringe filters before being subjected to liquid chromatography analysis. Triplicate extraction was performed on both samples.

### LC-ESI-MS Method

An Agilent 1290 Liquid Chromatography system (Agilent Technologies, CA, USA) coupled with a 6520 Q-TOF tandem mass spectrometer was used to separate compounds from the methanolic extracts of CO oil and oleoresin. The mass detector was a Q-TOF accurate mass spectrometer equipped with an electrospray ionization (ESI) interface and controlled by the MassHunter software. In brief, 2 μl of the sample solution comprising a mixture of compounds were loaded on a 2.1 mm (I.D.) Agilent Zorbax Eclipse Plus C-18 (100 mm in length) analytical column (with a particle size of 1.8 μm) that was maintained at 25°C. The analysis (positive mode) was performed by applying a gradient run that comprised mobile phase A (0.1% formic acid in water) and mobile phase B (100% acetonitrile with 0.1% formic acid), with a flow rate of 0.25 ml/min. As for the negative mode, the mobile phases used were mobile phase A (0.1% ammonium formate in water) and mobile phase B (100% acetonitrile).

Several trials were conducted to optimize the chromatographic conditions prior to the actual analysis by the application of different compositions and gradients of mobile phases. The gradient was run as follows: 5–90% B for 35.0 min, 90–90% B for 6 min, 90–5% for 0.1 min, and 5–5% for 6.9 min. The total run time for the LCMS analysis was 48 min, with a 13-min post-run time. The ionization conditions were adjusted to a capillary temperature of 300°C and 4000 voltage. The nebulizer pressure was 45 psi, and the nitrogen flow rate was 10 l/min. All mass spectrometry data were recorded in both positive and negative ion modes. The acquisition rate was 1.03 spectra/s across the ranges of 100–1000 *m/z* for both positive and negative ion modes.

Both CO samples were subjected to the LCMS Q-TOF in positive and negative ion modes for the profiling of the compounds or metabolites present in CO oil and oleoresin based on one replicate. Initially, the available plant metabolites were tentatively identified through the matching of masses of the compounds with the PlantCyc database. The MS mass tolerances were set between 0.05 and 0.1 Da. All the exact masses of unknown metabolites were further compared with the other online chemical databases (PubChem, LIPID MAPS, and ChemSpider), as well as some published scientific articles. The analysis was only focused on the electron ionization MS ranged between *m/z* 100 and *m/z* 1,000. The LCMS analysis was performed by the Proficient Lab Malaysia (Selangor, Malaysia) and the tentative identification of the compounds in the methanolic extracts of CO fat samples was done with the help of a chemist from the company.

### Fatty Acid Methyl Esters

Analysis of fatty acid methyl esters (FAMEs) was performed based on the IUPAC 2.301 protocol (IUPAC, 1987). Briefly, FAMEs were analyzed and determined using the Waters Gas Chromatography (GC) coupled with a flame ionization detector. The GC column used was Agilent DB-23 Capillary GC Column, 60 m × 0.25 mm, id 0.25 μm (J&W Scientific, Inc., CA, USA). The temperatures for injector and detector were 130 and 220°C, respectively. The nitrogen carrier gas flow was set at 0.53 ml/min. Fatty acid content in CO oil and oleoresin was quantified and determined based on the FAMEs.

## Results and Discussion

### LCMS Profiling and Tentative Identification of Potential Compounds

A sensitive, accurate, and specific method using high-performance liquid chromatography (HPLC) and ESI-MS was developed for the separation and tentative identification of compounds found in the semi-polar fraction (which consists of polar and non-polar compounds) of the fat samples. The molecular masses were assigned by ESI-MS. The subsequent structural characterization was carried out by a tandem mass spectrometric method. The accurate mass and fragmentation behavior of the compounds were investigated using ESI-Q-TOF in negative and positive ion modes.

Liquid chromatography-mass spectrometry (LCMS) combines the techniques of physical separation capabilities of liquid chromatography with mass analysis capabilities of mass spectrometry. This application is oriented toward the general detection and tentative identification of potential compounds in a plant mixture (Khoo et al., 2012). MS measures the mass-to-charge ratio of charged particles. It is used for determining masses of particles and the elemental composition of a sample or molecule, as well as for elucidating the chemical structure of molecules. Fatty acids, lipid metabolites, phenolic compounds, terpenoids, peptides, and other chemical components in the extracts of CO fat samples were tentatively identified based on the available plant metabolites and chemical databases.

### Fatty Acids and Other Lipids

As shown in Table 1, the identified fatty acids in CO oil and oleoresin were saturated fatty acids (SFAs), monounsaturated fatty acids (MUFAs), and polyunsaturated fatty acids (PUFAs). The SFAs (C8–C17) identified were caprylic acid (C8:0), lauric acid (C12:0), myristic acid (C14:0), palmitic acid (C16:0), and margaric acid (C17:0). Caprylic acid is a common eight-carbon fatty acid, which is abundantly found in coconut oil (Marina et al., 2009). Its systematic name is octanoic acid or caprylic acid. This fatty acid had a retention time of 19.14 min. Its anion was detected at *m/z* 143. Lauric acid, also known as dodecanoic acid, retained as peak 36 of the negative ionization mode, which had *m/z* 199 (**Figure 2**). Similar to palmitic acid and myristic acid, both of these compounds detected at the chromatogram of negative ionization mode, had MS base peak at *m/z* 255 and 227, respectively. However, palmitic acid was detected in the chromatogram of both ionization modes.

**Table 1:** LCMS profile (fatty acids and other lipids) of *Canarium odontophyllum* oil and oleoresin extracted with supercritical carbon dioxide.

**Peak**	**Identity**	**Molecular formula**	**Samples**		**t_**R**_ (min)**	**Monoisotopic mass found**	**Monoisotopic mass calculated**	**ESI mode**	**[M+H]^**+**^/[M–H]^**−**^**	**Relative abundance**
			**Oil**	**Oleoresin**						
2	Methyl capric acid	C_11_H_22_O_2_S	√	√	12.64	218.133	218.134	–	263 [M–H+46]^−^	328098.25
3	Isomer of trihydroxy-octadecenoic acid	C_18_H_34_O_5_	√	√	16.94	330.244	330.241	–	329	1633349.63
4	Isomer of trihydroxy-octadecenoic acid	C_18_H_34_O_5_	√	√	18.38	330.244	330.241	–	329	169859.73
5	Hydroperoxy-epoxy-octadecenoic acid	C_18_H_32_O_5_	√	√	19.10	328.228	328.225	–	327	462087.59
6	Octanoic acid (Caprylic acid)	C_8_H_16_O_2_	√	√	19.14	144.115	144.115	–	143	872324.88
4	Phytosphingosine	C_18_H_39_NO_3_	√	√	19.42	317.293	317.293	+	318	698409.69
8	Isomer of hydroxy-oxooctadeca-dienoic acid	C_18_H_30_O_4_	√	√	21.68	310.217	310.214	–	309	899460.31
9	Isomer of hydroperoxyocta-decadienoic acid	C_18_H_32_O_4_	√	√	21.86	312.233	312.230	–	311	1000000.0
10	Methyloctanoic acid	C_9_H_18_O_2_	√	√	21.96	158.131	158.131	–	157	1448654.38
6	Sphinganine	C_18_H_39_NO_2_	√		22.13	301.298	301.298	+	302	348809.94
11	Isomer of hydroxy-oxooctadeca-dienoic acid	C_18_H_30_O_4_	√	√	22.21	310.218	310.214	–	309	900802.31
12	Isomer of hydroperoxyocta-decadienoic acid	C_18_H_32_O_4_	√	√	22.40	312.234	312.230	–	311	1945015.75
13	Octadecenedioic acid	C_18_H_32_O_4_	√	√	22.65	312.234	312.234	–	311	1524944.88
8	Octadecenedioic acid	C_18_H_32_O_4_	√	√	22.79	312.229	312.234	+	313	53468.66
14	Epoxy-hydroxystearic acid	C_18_H_33_O_4_	√	√	22.89	314.248	314.246	–	313	2190024.75
15	Diglyceride	C_40_H_64_O_5_	√	√	23.21	624.473	624.475	–	311 2[M–H]^−^	2099296.25
10	Dimethyl 9-octadecenedioic acid	C_20_H_36_O_4_	√		23.23	340.261	340.261	+	341	136285.84
13	Diepoxylinoleic acid	C_18_H_32_O_4_	√		23.94	312.230	312.230	+	335 [M+H+22]^+^	246712.27
19	Isomer of fluoro-myristic acid	C_14_H_27_FO_2_	√	√	24.20	246.202	246.199	–	291 [M–H+46]^−^	970799.56
20	3-Hydroxymyristic acid	C_14_H_28_O_3_	√		24.44	244.206	244.204	–	243	439717.59
15	N,N-dimethyl-safingol	C_20_H_43_NO_2_	√	√	24.98	329.329	329.329	+	330	255155.25
23	Stearidonic acid	C_18_H_28_O_2_	√	√	25.15	276.211	276.209	–	275	576641.5
23	18:2–16:3-MGDG	C_43_H_72_O_10_	√	√	25.17	748.515	748.515	–	807 [M–H+60]^−^	753292.19
16	Stearidonic acid	C_18_H_28_O_2_	√	√	25.25	276.209	276.209	+	277	1001432.06
24	Oxo-oleic acid	C_18_H_32_O_3_	√	√	25.44	296.236	296.235	–	295	1027061.81
25	Oxo-hydroxy-octadecenoic acid	C_18_H_32_O_4_	√		25.44	312.232	312.230	–	311	643098.06
17	4-keto-9,11,13-octadecatrienoic (Licanic acid)	C_18_H_28_O_3_	√	√	25.48	292.204	292.204	+	293	194151.56
17	2-R-hydroperoxy-linolenic acid	C_18_H_30_O_4_	√	√	25.48	310.214	310.214	+	311	147534.75
26	Isomer of hydroxy-oxooctadeca-dienoic acid	C_18_H_30_O_4_	√	√	25.70	310.217	310.214	–	309	1518320.25
27	(+)-(12S,13R)-Epoxy-cis-9-octadecenoic acid (Vernolic acid)	C_18_H_32_O_3_	√	√	25.98	296.238	296.235	–	295	562747.63
18	17-Octadecene-9,11-diynoic acid (Isanic acid)	C_18_H_26_O_2_	√		26.05	274.193	274.193	+	275	113721.77
18	8-Hydroxyisanic acid	C_18_H_28_O_3_	√	√	26.07	292.203	292.204	+	293	104970.96
28	Isomer of fluoro-myristic acid	C_14_H_27_FO_2_	√	√	26.26	246.201	246.199	–	291 [M–H+46]^−^	301590.97
19	Dihydroxyisanic acid	C_18_H_30_O_4_	√	√	26.37	310.214	310.214	+	333 [M+H+22]^+^	47970.52
19	γ-Linolenic acid	C_18_H_30_O_2_	√	√	26.41	278.224	278.225	+	279	242436.94
29	Isomer of fluoro-myristic acid	C_14_H_27_FO_2_	√	√	26.44	246.200	246.199	–	291 [M–H+46]^−^	2254250.5
30	Octanoic acid, phenylmethyl ester	C_15_H_22_O_2_	√	√	26.78	234.164	234.162	–	233	462378.75
20	Vernolic acid	C_18_H_32_O_3_	√	√	27.10	296.235	296.235	+	319 [M+H+22]^+^	706011.94
20	α-Linolenic acid	C_18_H_30_O_2_	√	√	27.11	278.225	278.225	+	279	2399528.25
23	Colneleic acid (Conjugated linoleic acid)	C_18_H_30_O_3_	√	√	27.89	294.219	294.219	+	295	731395.06
24	Octadecadiynoic acid	C_18_H_28_O_2_	√	√	28.36	276.209	276.209	+	277	1170379.5
36	(E,E)-3,7,11-Trimethyl-2,6,10-dodecatrienyl decanoic acid	C_25_H_44_O_2_		√	28.97	376.336	376.334	–	421 [M–H+46]^−^	718831.19
36	Dodecanoic acid (Lauric acid)	C_12_H_24_O_2_	√	√	29.01	200.179	200.178	–	199	2459635.25
37	Octadecanedioic acid	C_18_H_33_O_4_	√	√	29.24	314.249	314.246	–	313	479845.78
26	Isomer of hydroxyoctadeca-dienoic acid	C_18_H_32_O_3_	√	√	29.64	296.235	296.235	+	297	1052650.5
38	1-18:1-2-16:1-monogalactosyl-diacylglycerol	C_43_H_78_O_10_	√	√	29.81	754.562	754.560	–	753 [M–H]^−^	558657.69
27	Isomer of hydroxyoctadeca-dienoic acid	C_18_H_32_O_3_	√	√	29.86	296.235	296.235	+	297	972653.25
28	3-methylbutyl octadecanoate triepoxidized	C_23_H_40_O_5_	√	√	30.42	396.286	396.287	+	397	25413.52
29	1-Monomyristin	C_17_H_34_O_4_	√	√	30.64	302.245	302.246	+	303	162373.84
30	2-Monolinolenin	C_21_H_36_O_4_	√	√	30.74	352.261	352.261	+	353	351743.47
31	1-Monopalmitolein	C_19_H_36_O_4_	√	√	31.71	328.261	328.261	+	329	283069.38
31	Linoleic acid	C_18_H_32_O_2_	√	√	31.74	280.240	280.240	+	281	302300.25
31	Hydroxyoleic acid	C_18_H_34_O_3_	√	√	31.74	298.250	298.251	+	299	196857.52
32	Heptadecanoic acid (Margaric acid)	C_17_H_34_O_2_	√		32.08	270.257	270.256	+	293 [M+H+22]^+^	380563.78
45	Triglyceride	C_61_H_104_O_6_	√	√	32.29	934.704	934.705	–	933	107065.19
33	2-Monolinolein	C_21_H_38_O_4_	√	√	32.43	354.277	354.277	+	355	1197577.25
46	Dihydroxystearic acid	C_18_H_36_O_4_	√	√	32.53	316.264	316.261	–	361 [M–H+46]^−^	555409.13
35	(+)-3-O-Myristoyl-L-1-isovalerin	C_22_H_42_O_5_		√	32.69	386.303	386.303	+	387	175619.13
47	Linoleic acid	C_18_H_32_O_2_	√	√	32.79	280.243	280.240	–	279	958292.0
36	Fragment of 1-linoleoyl glycerol	C_18_H_30_O	√	√	32.95	262.229	262.230	+	263	1713004.0
36	1-Monolinolein	C_21_H_38_O_4_	√	√	32.96	354.277	354.277	+	355	2410527.0
36	Oleoylethanol-amine	C_20_H_39_NO_2_	√		33.00	325.297	325.298	+	326	68761.23
48	Tetradecanoic acid (Myristic acid)	C_14_H_28_O_2_	√	√	33.14	228.211	228.209	–	227	2460460.75
37	Methyl 9,12-epoxy-9,11-octadecadienoic acid Methyl 9,12-epoxy-9,11-octadecadienoic acid	C_19_H_32_O_3_	√	√	33.35	308.235	308.235	+	309	197015.42
49	Triglyceride	C_62_H_94_O_6_	√	√	33.56	934.707	934.705	–	933	268820.31
38	Dihomo-γ-linolenic acid	C_20_H_34_O_2_	√	√	33.69	306.255	306.256	+	307	410266.31
50	Hexyldecanoic acid (Isopalmitic acid)	C_16_H_32_O_2_	√	√	33.89	256.242	256.240	–	255	338213.09
51	Hexyldecanoic acid (Isopalmitic acid)	C_16_H_32_O_2_	√	√	34.03	256.240	256.240	+	257	226114.44
51	PG(O-18:0/21:0)	C_45_H_91_O_9_P	√	√	34.06	806.640	806.640	–	805	139159.8
39	Hexadecenoic acid (Palmitoleic acid)	C_16_H_30_O_2_	√	√	34.13	254.225	254.226	+	255	129454.65
40	Oleoamide	C_18_H_35_NO	√	√	34.51	281.271	281.272	+	282	380287.63
52	Palmitic acid	C_16_H_32_O_2_	√	√	34.52	256.242	256.240	–	255	1194524.75
41	Palmitic acid	C_16_H_32_O_2_	√	√	34.63	256.240	256.240	+	257	1327739.5
41	Distearin	C_39_H_76_O_5_		√	34.66	624.532	624.569	+	313 2[M+H]^+^	2445253.5
54	Hexacosanedioic acid	C_26_H_50_O_4_	√	√	34.79	426.375	426.371	–	425	210845.73
42	1-Monoolein	C_21_H_40_O_4_	√	√	34.91	356.292	356.293	+	357	1208423.25
55	Cis-9-octadecenoic acid (Oleic acid)	C_18_H_34_O_2_	√	√	34.93	282.254	282.256	–	281	1008154.63
42	Heneicosanedioic acid	C_21_H_40_O_4_	√	√	34.93	356.292	356.293	+	379 [M+H+22]^+^	1208423.25
43	2-Monopalmitin	C_19_H_38_O_4_	√	√	35.06	330.277	330.277	+	331	423401.13
43	1-Monopalmitin	C_19_H_38_O_4_	√	√	35.09	330.277	330.277	+	331	1025944.38
57	Diglyceride	C_58_H_92_O_6_	√		35.18	884.686	884.689	–	883	122385.25
44	Cis-9-octadecenoic acid (Oleic acid)	C_18_H_34_O_2_	√	√	35.46	282.255	282.256	+	283	169262.47
44	Phospholipid	C_26_H_43_O_9_P	√	√	35.48	530.267	530.265	+	553 [M+H+22]^+^	32274.36
45	Isomer of linoleic acid	C_18_H_32_O_2_	√	√	35.59	280.240	280.240	+	281	140725.72
60	Coixenolide	C_38_H_70_O_4_	√	√	35.59	590.531	590.527	–	635 [M–H+46]^−^	244293.89
45	Diglyceride	C_62_H_94_O_6_	√	√	35.63	932.789	932.783	+	933	66596.27
45	Dihydroxylanosterol	C_30_H_52_O_3_	√	√	35.57	460.391	460.392	+	483 [M+H+22]^+^	89171.32
46	Lanosterol	C_30_H_48_O	√	√	35.72	424.370	424.371	+	425	1026837.31
46	Hydroxylanosterol	C_30_H_50_O_2_	√	√	35.71	442.381	442.381	+	443	1287918.88

**Figure 1:**
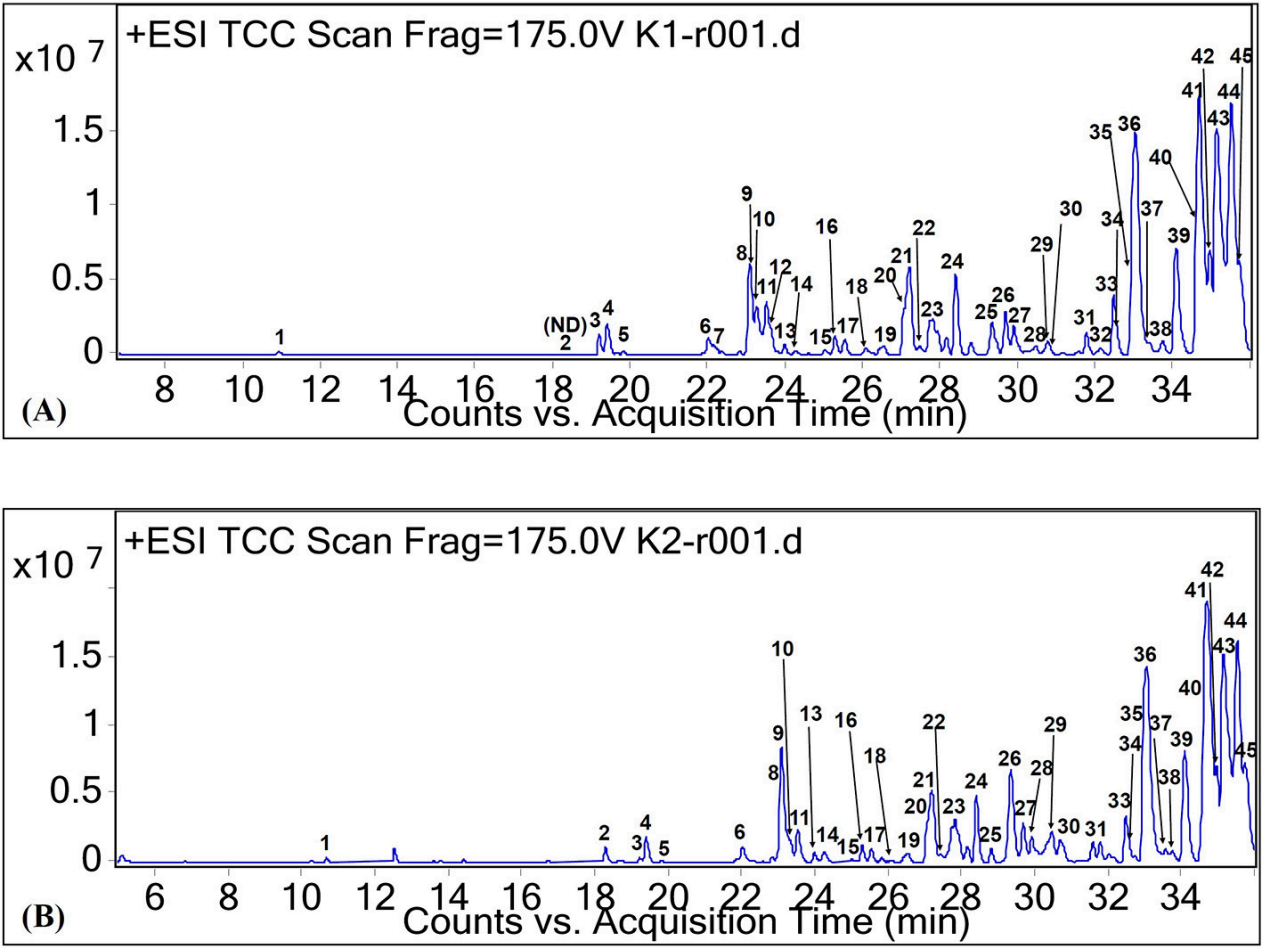
Total ion chromatograms of CO oil **(A)** and oleoresin **(B)** based on positive ionization mode.

Among the SFAs identified in this study, margaric acid was detected at RT of 32.08 min (peak 32, Figure 1). It is also known as heptadecanoic acid. The cation of this fatty acid was tentatively identified as *m/z* 293. It was only found in CO oil. All the other types of SFA were also tentatively identified in both CO oil and oleoresin. Although margaric acid seldom occurs in vegetable fat, it was found in CO oil at low concentration (based on the relative abundance value).

In this study, hexanoic acid (caproic acid, C6:0), decanoic acid (capric acid, C10:0), and octadecanoic acid (stearic acid, C18:0) were not detected in both CO oil and oleoresin. Lauric acid and myristic acid were the major fatty acids in CO oil and oleoresin, with relative abundance values higher than 2.45 × 10^6^ unit. Lauric acid is also considered a medium-chain fatty acid.

On the other hand, the major MUFAs which were tentatively identified in both CO oil and oleoresin are palmitoleic acid ([M+H]^+^ at *m/z* 255) and oleic acid ([M+H]^+^ at *m/z* 283). As shown in Figure 1, both of these MUFAs were detected in the two fat samples with RT of 34.13 min (peak 39) and 35.46 min (peak 44). Palmitoleic acid (9-hexadecenoic acid) is an omega-7 MUFA with molecular formula C_16_H_30_O_2_. It is an important fatty acid for the human body as it is made up of 5.6% of human epidermis (Carruthers, 1964). Besides the C16 MUFA, oleic acid (octadecenoic acid) was another MUFA found in the CO oil and oleoresin. Oleic acid in its cis-9 form was tentatively identified in the chromatograms of both positive and negative ionization modes, with the electron ionization MS of both cation and anion at *m/z* 283 and 281, respectively. In fact, cis-9-oleic acid is the common fatty acid in plant oil (D'Evoli et al., 2015). Oleic acid is a C18 MUFA with various applications in the food industry and plays an important role in lipase synthesis. It has been reported to be able to induce lipase production during food processing, especially in the dairy industry (Aravindan et al., 2007).

In this study, we tentatively identified six PUFAs and other unclassified fatty acids. Among the naturally occurring PUFAs, linoleic acid (peak 31, Figure 1), α-linolenic acid (peak 20, Figure 1), γ-linolenic acid (peak 19, Figure 1), di-homo-γ-linolenic acid (peak 38, Figure 1), and stearidonic acid (peak 16, Figure 1) were tentatively identified in both CO oil and oleoresin. For the comparison between α-linolenic acid and γ-linolenic acid, a higher relative abundance value was observed for α-linolenic acid than the value observed for γ-linolenic acid. Besides the relative abundance values, γ-linolenic acid eluted at 0.7 min faster than α-linolenic acid. Based on the report by Momchilova and Nikolova-Damyanova (2003), the elution of fatty acid with more than one double bond in the longer carbon chain, which held itself stronger than the shorter chain. Therefore, a γ-linolenic acid which had the double bond with a shorter neighboring carbon chain (26.41 min) eluted faster than α-linolenic acid with a longer carbon chain (27.11 min).

The other PUFAs identified were C18:2n6, C18:2n6, C18:2, C18:3n3, C18:3n6, C20:3n6, and C18:4n3. Linoleic acid was tentatively identified based on [M+H]^+^ and [M–H]^−^ at *m/z* 281 and *m/z* 279. Both α- and γ-linolenic acid had [M+H]^+^ at *m/z* 279. This study shows that linolenic acid in its alpha form was the major compound with a high relative abundance value (Table 1), but the gamma form is one of the minor fatty acids in both CO oil and oleoresin. Di-homo-γ-linolenic acid (C_20_H_34_O_2_) is the PUFA with longer carbon-chain; it was found to have [M+H]^+^ at *m/z* 307. Stearidonic acid is another omega-3 fatty acid found in both CO oil and oleoresin. It retained at 25.2 ± 0.05 min of the chromatograms of both positive and negative ion modes. It had a monoisotopic mass of 276 Da.

**Figure 2:**
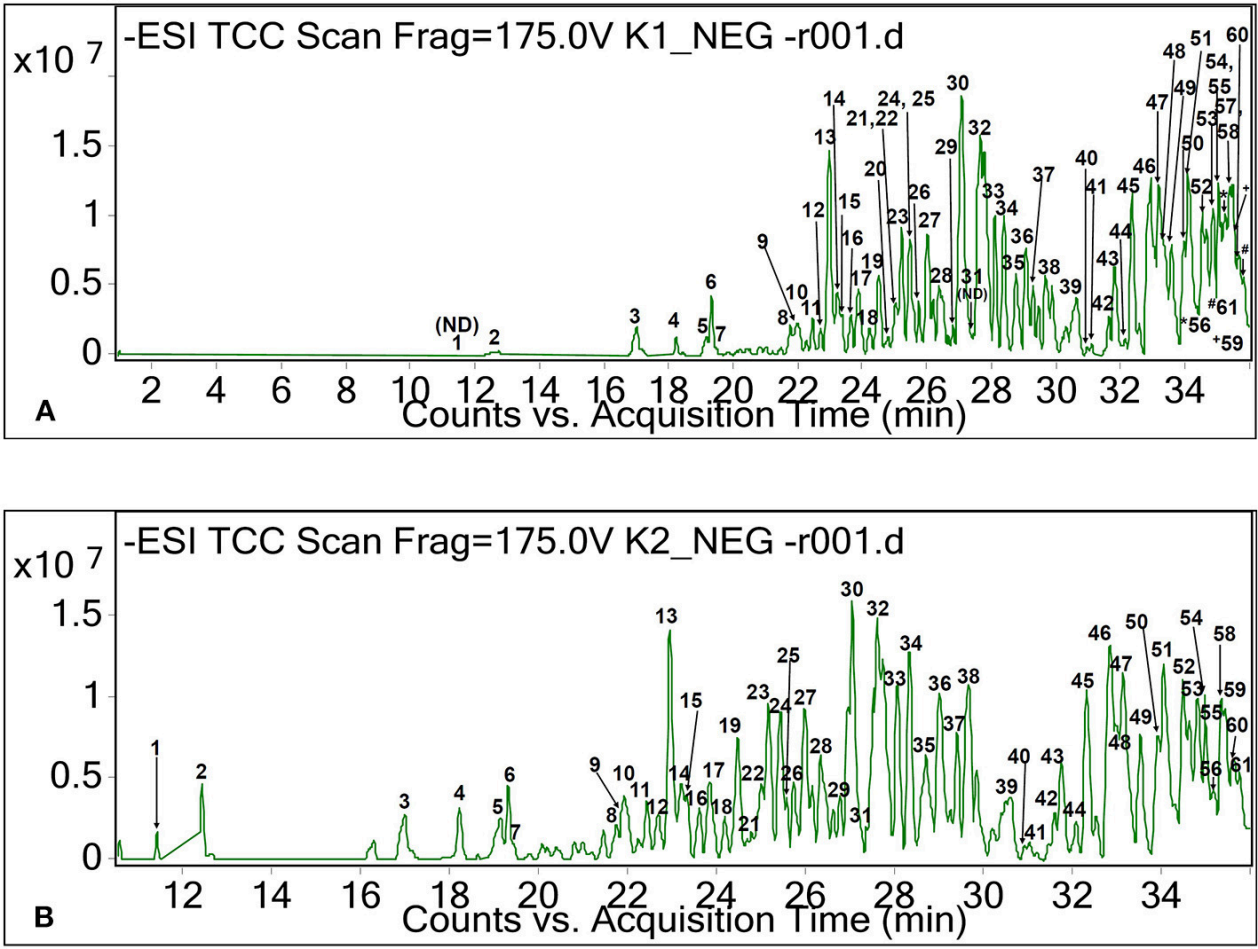
Total ion chromatograms of CO oil **(A)** and oleoresin **(B)** based on negative ionization mode.

Besides these main fatty acids, their isomers, hydroxylated, dihydroxylated, trihydroxylated, and epoxylated forms of fatty acids, as well as other chemical conformations, were tentatively identified for the CO oil and oleoresin. As shown in Table 1, these compounds had [M+H]^+^ and [M–H]^−^ at between *m/z* 157 and 421. Methyloctanoic acid (C_9_H_18_O_2_, monoisotopic mass of 158 Da, peak 10 of Figure 2) was the methylated fatty acid with the least molecular weight, whereas (E,E)-3,7,11-trimethyl-2,6,10-dodecatrienyl decanoic acid (peak 36, Figure 2) was a large compound, and it has capric acid as its base unit. This compound was only detected in CO oleoresin. Although capric acid (decanoic acid) was not detected in the fat samples, our finding shows that it existed in methylated forms. Due to the data being obtained from the anionic and cationic masses, we were not able to determine the position of all methylated fatty acids. Besides the anion of peak 36, (+)-3-O-myristoyl-L-1-isovalerin (peak 35, [M+H]^+^ at *m*/*z* 387) was detected only in CO oleoresin. We concluded that only two lipids were specifically detected in CO oleoresin. In addition to these findings, the results show that the identified monoglycerides and diglycerides were part of the fat components in CO oil and oleoresin.

As shown in Figure 1 and Table 1, other lipids including phytosphingosine (peak 4, RT 19.42 min, [M+H]^+^ at *m/z* 318), dimethyl-safingol (peak 15, RT 24.98, [M+H]^+^ at *m/z* 330), and phospholipid (peak 44, RT 35.48 min, [M−22]^+^ at *m/z* 553) were also tentatively identified in the oil and oleoresin. Due to the tentative identification of lipids being performed based on monoisotopic masses, we could not identify the type of phospholipid detected in the fat samples. Nevertheless, sphinganine (peak 6 of Figure 1, RT 22.13 min, [M+H]^+^ at *m/z* 302) was detected in CO oil. Based on the relative abundance values of the MS spectra, these lipids were the minor compounds detected in the CO oil and oleoresin.

In this study, lauric acid, myristic acid, α-linolenic acid, dihydroxystearic acid, and epoxy-hydroxystearic acid were relatively abundant in the fat samples. These compounds can be considered as the major fatty acids in CO oil and oleoresin due to their high relative abundance values. Based on these findings, we could conclude that CO oil and oleoresin have a balanced fatty acid composition, with almost equal levels of SFA, MUFA, and PUFA. The relative abundance values also proved that major fatty acids were tentatively identified as methyloctanoic acid (a methylated form of caprylic acid), palmitic acid, oleic acid, oxo-oleic acid, trihydroxyoctadecenoic acid, hydroperoxyoctadecadienoic acid, hydroxy-oxo-octadecadienoic acid, octadecenedioic acid, stearidonic acid, octadecadiynoic acid, and heneicosanedioic acid. The levels of these long-chain fatty acids especially epoxy-hydroxystearic acid and dihydroxystearic acid were found to be in a higher amount in CO oleoresin than in CO oil. The result shows that the level of dihydroxystearic acid was 2.35 times higher in CO oleoresin than in the oil. These long-chain fatty acids might contribute to the high viscosity of CO oleoresin compared to that of the CO oil.

Monoolein (C_21_H_40_O_4_, [M−22]^+^ at *m/z* 357, peak 42 of Figure 1), monopalmitin (C_19_H_38_O_4_, [M+H]^+^ at *m/z* 331, peak 43 of Figure 1), and monolinolein (C_21_H_38_O_4_, [M+H]^+^ at *m/z* 355, peaks 33 and 36 of Figure 1), as well as diglyceride (C_40_H_64_O_5_, [M−2H]^−^2 at *m/z* 311, peak 15 of Figure 2) were detected as the major fat components in the fat samples. The result also showed that 1-monolinolein (peak 36 of Figure 1) was the only major glyceride in the fat samples. Monoolein was also found to be relatively abundant in the fat samples. In addition to these fatty acids, lanosterol and its hydroxyl forms were tentatively identified in both of the CO fat samples. As shown in Table 1, at RT 35.7 min, lanosterol (C_30_H_48_O, peak 46 of Figure 1) and its hydroxyl-forms (peaks 45 and 46) were the major plant sterols in the CO fruit.

Five diglycerides were detected in both fat samples (Table 1). Although these diglycerides could have several chemical conformations, lipid with the masses found may only correspond to the structures of diglycerides as suggested in the LIPID MAPS database. According to the database from LIPID MAPS, a lipid with monoisotopic mass of 624 Da and the formula of C_40_H_64_O_5_ may correspond to DG(17:2(9Z,12Z)/20:5(5Z,8Z,11Z,14Z,17Z) or DG(15:1(9Z)/22:6(4Z,7Z,10Z,13Z,16Z,19Z). The LIPID MAPS database also suggested that anions of peaks 45 and 49 could be triglycerides. However, only one of these diglycerides was found in the CO oil, which had the molecular formula of C_58_H_92_O_6_ ([M–H]^−^ at *m/z* 884.7, peak 57 of Figure 2). The molecular structure of this glyceride was unknown.

A wide range of fatty acids was determined in the oil and oleoresin of the CO fruit. Some of these fatty acids have either positive or negative effects on human health. CO oil also has a balanced amount of both saturated and unsaturated fatty acids. Saturated fatty acids are known to have detrimental effects, especially in terms of increased risk of cardiovascular diseases and stroke (Siri-Tarino et al., 2010). Among the different lengths of saturated fatty acids, medium-chain triglycerides have protective effects against cardiovascular diseases and many other diseases.

Lauric acid is one of the medium-chain triglycerides that was detected in the CO fat samples in a relatively high amount. Previous studies showed that a diet rich in lauric acid is less likely to promote obesity. Lauric acid was also found to be able to improve the lipid profile from the experimental trials (McCarty and DiNicolantonio, 2016). As compared to lauric acid (C12), myristic acid (C14) is considered as the long-chain fatty acid. Although myristic acid has only two carbon chains longer than those of lauric acid, it poses deleterious effects on human health. Same with palmitic acid, a diet rich in myristic acid could increase the risk of cardiovascular diseases following the rise of LDL-cholesterol and apolipoprotein B levels in healthy men and women (Zock et al., 1994).

Previous studies reported that monoglycerides and diglycerides had protective effects against several chronic diseases (Feltes et al., 2013). Due to the anti-diabetic and antiatherogenic effects of monoolein (Cho et al., 2010), the high level of monoglyceride in CO oil and oleoresin makes these fat samples as potential functional foods. Diglyceride is a prospective therapeutic agent for antiobesity. The effect is probably due to the increased β-oxidation of the fatty acids found during the metabolism of the diglyceride (Lo et al., 2008).

In addition to the balance fatty acid composition, the CO oil has a low level of linoleic acid. The level of linoleic acid in human blood is the key to the development of several chronic diseases (Spiteller, 1998). Therefore, a balanced fatty acid composition of the vegetable oil is essential for maintaining good health besides providing oxidative stability.

In this study, we found a desirable level of isanic acid in CO oil and oleoresin. Isanic acid, also known as erythrogenic acid (C_18_H_26_O_2_), was tentatively identified as peak 18 (Figure 1). It had protonated molecular ion [M+H]^+^ at *m/z* 275, and it was detected only in CO oil. However, 8-hydroxyisanic acid (C_18_H_28_O_3_) was found in both of the CO fat samples, with the RT of 26.07 min (Table 1). Isanic acid is also a less common fatty acid found in vegetable oil. It had been first detected in *Ongokea gore* seed (isano) oil from African countries (Ntumba et al., 2015). It is known as a non-edible fatty acid. The literature showed that isanic acid turns to a vivid red when exposed to light at increasing temperature (Black and Weedon, 1953). It also contributes to the color of the oil besides carotenoids. Although isanic acid was detected in the CO oil, our previous study proved that the oil is not hepatotoxic (Faridah Hanim et al., 2015).

Based on the FAMEs analysis, the main component of CO oil was palmitic acid (41.53 ± 0.13%), followed by oleic acid (38.79 ± 0.01%), and (cis) linoleic acid (11.95 ± 0.01%). The fatty acid composition of the CO oil was quantified and tabulated in Table 2. CO oil was characterized as SFA-rich oil due to its high SFA composition (47.62 ± 0.13%). However, the MUFA and PUFA in CO oil cannot be disregarded. CO oil contained 39.93 ± 0.16 % MUFA and 12.48 ± 0.01% PUFA. Among the SFA, no short-chain fatty acids were detected in the oil.

**Table 2:** Fatty acid composition of *Canarium odontophyllum* oil extracted with supercritical carbon dioxide.

**Carbon**	**FAMEs**	**% in fat**	**mg/100 g**
C8	Caprylic	0.05 ± 0.00	47.94 ± 2.00
C10	Capric	0.01 ± 0.00	5.99 ± 0.14
C11	Undecanoic	0.01 ± 0.01	9.50 ± 6.76
C12	Lauric	0.72 ± 0.07	694.46 ± 38.38
C13	Tridecanoic	0.00 ± 0.00	3.81 ± 0.66
C14	Myristic	0.28 ± 0.00	270.41 ± 10.39
C15	Pentadecanoic	0.03 ± 0.00	25.42 ± 1.27
C16	Palmitic	41.53 ± 0.13	40273.79 ± 1517.22
C17	Heptadecanoic	0.11 ± 0.00	108.37 ± 5.49
C18	Stearic	4.31 ± 0.01	4175.36 ± 167.51
C20	Arachidic	0.10 ± 0.00	93.08 ± 4.36
C21	Henicosanoic	0.02 ± 0.01	18.51 ± 11.23
C22	Behenic	0.25 ± 0.07	245.22 ± 77.32
C23	Tricosanoic	0.11 ± 0.00	106.69 ± 1.89
C24	Lignoceric	0.10 ± 0.01	97.12 ± 16.33
Total SFA		47.62 ± 0.13	46175.65 ± 1746.62
C14:1	Myristoleic	0.04 ± 0.00	40.51 ± 0.3
C15:1	Cis-10-pentadecenoic	0.04 ± 0.00	34.59 ± 0.97
C16:1	Palmitoleic	0.64 ± 0.01	615.88 ± 24.31
C17:1	Cis-10-heptadecanoic	0.03 ± 0.00	25.20 ± 0.97
C18:1n9c	Oleic	38.79 ± 0.01	37616.98 ± 1541.0
C20:1n9	Cis-11-eicosenoic	0.07 ± 0.01	29.41 ± 41.58
C22:1n9	Erucic	0.04 ± 0.03	39.27 ± 27.63
C24:1	Nervonic	0.30 ± 0.13	289.66 ± 146.71
Total MUFA		39.93 ± 0.16	38691.48 ± 1783.48
C18:2n6c	Linoleic (cis)	11.95 ± 0.01	11593.46 ± 456.69
C18:3n6	γ-linolenic	0.11 ± 0.00	103.92 ± 1.88
C18:3n3	α-linolenic	0.40 ± 0.00	387.11 ± 15.2
C20:4n6	Arachidonic	0.02 ± 0.01	14.71 ± 4.12
Total PUFA		12.48 ± 0.01	12099.20 ± 477.88

The saturated carbon chains detected were C8, C11, and C12. These carbon chains were determined as caprylic acid, undecanoic acid, and lauric acid, as well as long carbon chains C14-C24. Based on the carbon chains, these fatty acids should be known as long-chain fatty acids. The fatty acids were named as follows: myristic acid (C14), pentadecanoic acid (C15), palmitic acid (C16), heptadecanoic acid (C17), stearic acid (C18), arachidic acid (C20), heneicosanoic acid (C21), behenic acid (C22), tricosanoic acid (C23), and lignoceric acid (C24).

Based on the results obtained, the SFA composition in the CO oil was mainly long-chain fatty acids. Although some of the carbon chains such as C15, C17, C21, and C23 are rarely found in fruit oils, these fatty acids were detected based on the carbon chains. Therefore, LCMS analysis was performed to confirm the existence of these fatty acids. The LCMS data showed that the fatty acids with carbon chains of C20–C24 were not detected in both CO oil and oleoresin. These carbon chains could be derived from the other types of lipid or hydrocarbon-containing compounds.

### Phenolics and Terpenoids

The phenolic compounds identified in most of the fat and oil were in semi-polar to non-polar forms. In this study, we managed to tentatively identify semi-polar coumaric acid and anisic acid as the main phenolic acids in CO oil and oleoresin. Although the relative abundance values of these phenolic acids were not as high as those observed in the major fatty acids, a low amount of these compounds has a good effect on human health because phenolics are strong antioxidants. Peak 3 was tentatively identified as the peak consisting of a mixture of flavonoids at RT of 19.36 min (Table 3; Figure 1), including prenyl-naringenin, sophoraflavanone B, and neobavaisolavone. The anions of these compounds had electron ionization mass spectra at *m/z* 340, 340, and 322 respectively. Peak 3 was also tentatively identified as either matairesinol or pinoresinol. These phenolics were the minor compounds tentatively identified in both of the fat samples.

**Table 3:** LCMS profile (phenolics and terpenoids) of *Canarium odontophyllum* oil and oleoresin extracted with supercritical carbon dioxide.

**Peak**	**Identity**	**Molecular formula**	**Samples**		**t_**R**_** **(min)**	**Monoisotopic mass found**	**Monoisotopic mass calculated**	**ESI mode**	**[M+H]^**+**^/[M–H]^**−**^**	**Relative abundance**
			**Oil**	**Oleoresin**						
**PHENOLIC COMPOUNDS**										
3	Matairesinol	C_20_H_22_O_6_	√	√	19.35	358.141	358.142	+	381 [M+H+22]^+^	172492.03
3	Pinoresinol	C_20_H_22_O_6_	√	√	19.35	358.141	358.142	+	381 [M+H+22]^+^	172492.03
3	8-Prenyl-naringenin	C_20_H_20_O_5_	√	√	19.36	340.131	340.131	+	323 [M–H2O]^+^	706622.94
3	Sophoraflavanone B	C_20_H_20_O_5_	√	√	19.36	340.131	340.131	+	323 [M+H–H2O]^+^	706622.94
3	Neobavaisolavone	C_20_H_18_O_4_	√	√	19.36	322.120	322.121	+	323	706622.94
10	Coumaric acid	C_9_H_8_O_3_	√	√	23.24	164.047	164.047	+	165	681723.69
11	Caffeoylquinic acid	C_16_H_18_O_9_	√	√	23.51	354.097	354.095	+	355	117188.53
14	Olivetol	C_11_H_16_O_2_	√	√	24.21	180.115	180.115	+	181	185940.86
21	Anisic acid (Methoxybenzoic acid)	C_17_H_26_O_3_	√	√	24.77	278.191	278.188	–	277	412985.5
43	Octacosyl caffeate	C_37_H_64_O_4_	√	√	31.75	572.482	572.481	–	617 [M–H+46]^−^	184760.38
**TERPENOIDS**										
1	Lacinilene C	C_15_H_18_O_3_		√	13.76	246.125	246.126	+	247	161711.34
3	Phaseollin	C_20_H_18_O_4_	√	√	19.36	322.120	322.121	+	323	706622.94
7	Cincassiol B	C_20_H_32_O_8_	√		22.22	400.209	400.210	+	401	26966.11
32	Bixin aldehyde	C_24_H_28_O_2_	√	√	27.72	348.209	348.209	–	347	282460.81
22	Kauralexin A2	C_20_H_30_O_8_	√	√	27.75	334.214	334.214	+	335	412137.34
33	Oleanonic aldehyde	C_30_H_46_O_2_	√	√	32.42	438.349	438.350	+	439	129427.81
34	Glycyrrhet-aldehyde	C_30_H_46_O_3_	√	√	32.54	454.345	454.35	+	455	32258.1
38	Oleanolic acid	C_30_H_48_O_3_	√	√	33.69	456.360	456.360	+	457	69752.61
58	Isomer of hydroxyl-apocarotenal	C_27_H_36_O_2_	√	√	35.30	392.272	392.272	–	391	603699.25
59	Isomer of hydroxyl-apocarotenal	C_27_H_36_O_2_	√	√	35.41	392.272	392.272	–	391	547084.19

As shown in Table 3, triterpenes, sesquiterpenes, and apocarotenoids were tentatively identified in the CO oil and oleoresin. Based on the relative abundance value of the peak, a moderate level of hydroxy-apocarotenal (C_27_H_36_O_2_) was retained at 35.3–35.4 min (Figure 2) with [M–H]^−^ at *m/z* 391 (Table 3). It is one of the carotenoids found in fruits (Gross and Eckhardt, 1981). Peaks 58 and 59 of the chromatogram of negative ionization mode (Figure 2) for both CO oil and oleoresin were tentatively identified as two isomers of hydroxy-apocarotenal. On the other hand, cincassiol B (C_20_H_32_O_8_) was only determined in CO oil. It gave a protonated molecular ion [M+H]^+^ at *m/z* 401 (peak 7, Figure 1). Peak 1 was tentatively identified only in the CO oleoresin as lacinilene C (C_15_H_18_O_3_) with RT 13.76 min and [M+H]^+^ at *m/z* 247. Also, phaseollin and kauralexin A2 together with oleanolic acid and oleanonic aldehyde were the terpenoids identified in both fat samples (Table 3).

The yellow hue of CO oil could be contributed by both apocarotenal and isanic acid. A previous study reported that apocarotenal has provitamin A activity (Bagdon et al., 1962). It is considered an intermediary of the conversion of β-carotene to vitamin A. Although the provitamin A activity is not as high as β-carotene, consumption of CO may improve the plasma vitamin A status of a healthy individual. Prasad et al. (2011) also reported that a moderate amount of β-carotene was determined in the saponified extract of CO fruit. In addition to the apocarotenal, carotenoids were not identified in the CO fat due to several reasons, one of which is that carotenoids occur in the plant as carotenoid-protein complexes and carotenoid glycosides (Jürgens and Weckesser, 1985).

### Volatiles and Other Aromatic Compounds

Table 4 shows the volatiles and other aromatic compounds identified in the CO oil and oleoresin. The use of LCMS in detecting volatiles causes a loss of most known volatiles in the fat samples. The commonly known volatiles identified in both CO oil and oleoresin were cuparene ([M+H]^+^ at *m/z* 202) and hexadecenal ([M+H]^+^ at *m/z* 238). In this study, 4-(2-hydroxy-propoxy)-3,5-dimethylphenol ([M+H]^+^ at *m/z* 196) and 1,2-dioctanoyl-1,2,3-butanetriol ([M+H]^+^ at *m/z* 358) were tentatively identified as the novel volatiles in CO oil. The volatiles were not detected in CO oleoresin. In addition to volatiles, other aromatic compounds ([M+H]^+^) identified in the fat samples were deoxyshikonin (*m/z* 273, peak 10), 4-prenylphlorisovalerophenone (*m/z* 279, peak 25), demethylphylloquinol (*m/z* 439, peak 33), and demethylphylloquinone (*m/z* 437, peak 34), found based on positive ionization mode (Table 4). The other quinol-based compounds identified were also not found in most of the commercial fruits.

**Table 4:** LCMS profile (volatiles and other aromatic compounds) of *Canarium odontophyllum* oil and oleoresin extracted with supercritical carbon dioxide.

**Peak**	**Identity**	**Molecular formula**	**Samples**		**t_**R**_** **(min)**	**Monoisotopic mass found**	**Monoisotopic mass calculated**	**ESI mode**	**[M+H]^+^**	**Relative abundance**
			**Oil**	**Oleoresin**						
**VOLATILES**										
1	4-(2-Hydroxy-propoxy)-3,5-dimethylphenol	C_11_H_38_O_3_	√		10.91	196.110	196.110	+	197	202387.48
10	1,2-Dioctanoyl-1,2,3-butanetriol	C_20_H_38_O_5_	√		23.22	358.272	358.272	+	381 [M+H+22]^+^	345105.97
22	(–)-Cuparene	C_15_H_22_	√	√	27.43	202.172	202.172	+	203	510508.72
41	(2E)-Hexadecenal	C_16_H_30_O	√	√	34.63	238.229	238.230	+	239	289036.38
**OTHER AROMATIC COMPOUNDS**										
10	Deoxyshikonin	C_16_H_16_O_4_	√	√	23.24	272.104	272.105	+	273	123640.84
25	4-Prenylphloriso-valerophenone	C_16_H_22_O_4_	√	√	29.29	278.152	278.152	+	279	26268.41
33	Demethyl-phylloquinol	C_30_H_46_O_2_	√	√	32.42	438.349	438.350	+	439	129427.81
34	Demethyl-phylloquinone	C_30_H_44_O_2_	√	√	32.54	436.334	436.334	+	437	69870.93

Literature shows that more than 30 volatiles were tentatively identified in the fruit of *Canarium pimela* (Lv et al., 2014). The fruit is from the genus *Canarium*, which should have a similar characteristic as found in the CO fruit. However, the volatiles found in the samples were far lesser than the volatiles reported in the previous study. It could be due to the loss of most of the volatiles in the sample during the SCO_2_ extraction of the CO oil. Therefore, only cuparene and hexadecenal were the major volatiles retained in the fat samples. This observation is supported by the finding from a previous study, where supercritical fluid extracted essential oils had lower percentages of volatiles than the essential oils extracted using simultaneous distillation-extraction apparatus (Díaz-Maroto et al., 2002).

Deoxyshikonin is one of the naphthoquinones found in plant. It has a molecular mass of 272 Da with a formula structure of C_16_H_16_O_4_. It has never been reported in fruit, except roots and barks. Therefore, deoxyshikonin is the first to be identified in the fruit of *Canarium*. According to Song (2015), valerophenone is the metabolite produced during strawberry ripening. Besides valerophenone, demethylphylloquinone is a fat-soluble compound identified in the CO fat. It is another form of a fat-soluble vitamin, also known as vitamin K, which is present naturally in most plants. The occurrence of these quinol-based compounds may add value to the CO oil and oleoresin.

### Peptides, Other Phytochemicals, and Unknown

In this study, we tentatively identified a few peptide fragments in the CO oil and oleoresin. These peptide fragments had monoisotopic masses of 340.2–414.2 Da (Table 5). Trp-Ala-Pro, His-Met-Lys, and Phe-Asn-Ser were tentatively identified in both of the CO fat samples. These peptides had molecular ions [M+H]^+^ at *m/z* 373, [M+H]^+^ at *m/z* 415, and [M–H]^−^ at *m/z* 365 respectively. However, Lys-Lys-Lys ([M+H]^+^ at *m/z* 403) and Pro-Lys-Pro ([M–H]^−^ at *m/z* 339) were only identified in CO oil and oleoresin, respectively. Peptides with the molecular weights of higher than 1,000 Da were not able to be identified in the oil samples. It is due to our mass searching technique which was based only on the PlantCyc database and some major online chemical databases. Nevertheless, we had no access to the database for peptide sequences. Therefore, the peptides with more than three amino acids and polypeptides remained unknown. This study makes way for other researchers to work on the peptide sequencing for CO oil.

**Table 5:** LCMS profile (peptides, other compounds, and unknowns) of *Canarium odontophyllum* oil and oleoresin extracted with supercritical carbon dioxide.

**Peak**	**Identity**	**Molecular formula**	**Samples**		**t_**R**_** **(min)**	**Monoisotopic mass found**	**Monoisotopic mass calculated**	**ESI mode**	**[M+H]^**+**^/[M–H]^**−**^**	**Relative abundance**
			**Oil**	**Oleoresin**						
**PEPTIDES**										
5	Trp-Ala-Pro	C_19_H_24_N_4_O_4_	√	√	19.79	372.179	372.180	+	373	51996.54
9	His-Met-Lys	C_17_H_30_N_6_O_4_S	√	√	23.06	414.204	414.205	+	415	2391460.25
17	His-Met-Lys	C_17_H_30_N_6_O_4_S	√	√	23.51	414.209	414.205	–	459 [M–H+46]^−^	498724.88
12	Lys-Lys-Lys	C_18_H_38_N_6_O_4_	√		23.59	402.293	402.295	+	425 [M+H+22]^+^	97721.0
32	Phe-Asn-Ser	C_16_H_22_N_4_O_6_	√	√	27.71	366.154	366.152	–	411 [M–H+46]^−^	218282.23
45	Pro-Lys-Pro	C_16_H_28_N_4_O_4_		√	35.61	340.211	340.211	–	339	175289.47
**OTHER COMPOUNDS**										
2	Hercynine	C_9_H_16_N_3_O_2_		√	18.27	198.125	198.124	+	199	509599.09
31	10-(β-Dimethyl-aminopropionyl)phenothiazine	C_17_H_18_N_2_OS		√	27.02	298.114	298.114	–	297	133534.45
39	Dodecyl hydroxy-phosphinate	C_25_H_36_NO_3_	√	√	30.59	398.272	398.269	–	397	2308994.0
41	Dodecenyl acetate	C_14_H_26_O_2_	√	√	30.91	226.195	226.193	–	225	578895.94
47	Tridecenyl acetate	C_15_H_28_O_2_	√	√	32.84	240.211	240.209	–	239	924669.44
59	(2S,3S)-2-(dicyclohexylmethyl)-3-hydroxypiperidin-1-ium	C_18_H_34_NO	√	√	35.37	280.264	280.264	–	279	2460187.5
60	4-{2-[4-(2-methylpropyl)piperazine-1-sulfonyl]ethyl}piperazin-1-ium	C_14_H_31_N_4_O_2_S	√	√	35.61	319.217	319.217	–	364 [M–H+46]^−^	493911.53
61	(S)-2,6-Bis(dibenzylamino)hexan-1-ol	C_34_H_41_N_2_O	√	√	35.74	493.322	493.322	–	538 [M–H+46]^−^	34379.78
**UNKNOWN COMPOUNDS**										
1	Unknown	C_18_H_20_NO_3_		√	11.40	298.145	298.144	–	297	480810.94
7	Unknown	C_17_H_26_O_6_S	√		19.28	358.146	358.145	–	357	678045.94
16	Unknown	–	√	√	23.30	404.281	–	–	403	329898.94
18	Unknown	C_32_H_66_N_6_OS_3_	√	√	23.82	646.446	646.446	–	645	272059.03
22	Unknown	C_11_H_34_N_10_O_4_	√	√	24.96	370.276	370.276	–	415 [M–H+46]^−^	625019.38
23	Unknown	C_37_H_40_FN_3_O_2_	√	√	25.17	577.312	577.311	–	576	258103.25
21	Unknown	C_15_H_22_	√	√	27.43	202.172	202.172	+	203	510508.72
33	Unknown	–	√	√	28.19	538.341	–	–	537	212423.05
34	Unknown	C_18_H_34_O_3_	√	√	28.32	298.255	298.251	–	297	1153108.38
35	Unknown	C_18_H_30_O_3_	√	√	28.73	294.226	294.219	–	293	2179957.0
40	Unknown	C_19_H_35_FNO_4_	√	√	30.71	360.255	360.255	–	359	314079.25
42	Unknown	C_21_H_32_N	√	√	31.44	298.253	298.254	–	297	349935.97
44	Unknown	C_21_H_32_N	√	√	32.08	298.254	298.254	–	297	937098.44
53	Unknown	C_16_H_32_F_2_N_3_O	√	√	34.61	320.251	320.251	–	365 [M–H+46]^−^	449587.53
56	Unknown	–	√	√	35.07	560.791	–	–	279 2[M–H]^−^	1560599.25

In nature, peptides are short-chain-linked amino acids with specific functions. The shortest peptide consists of two amino acids, whereas the peptide chain can be as long as a chain of 50 amino acids. In fact, peptide sequencing is typically performed using MALDI-MS. In this study, the three amino acid peptides were tentatively identified. Although these identified peptides do not have known functional properties, peptides have been reported to possess protective effect against *in vitro* oxidative stress (Ioudina and Uemura, 2003).

Besides peptides, eight other known compounds were tentatively identified in the fat samples (Table 5). The results showed that hercynine ([M+H]^+^ at *m/z* 198) and 10-(β-dimethyl-aminopropionyl)phenothiazine ([M–H]^−^ at *m/z* 298) were only found in the CO oleoresin. These compounds are the novel compounds identified in the fruit oil. Hercynine is the intermediate compound of ergothioneine biosynthesis in fungus (Askari and Melville, 1962). The other six organic compounds detected in the CO fat had [M–H]^−^ ranged between *m/z* 226 and 493 (Table 5), which include the derivatives of two heterocyclic amines. One of these six compounds, dodecyl hydroxyphosphinate (peak 39 of Figure 2), was a novel fatty acid derivative that was abundantly found in the fat samples. It was also one of the major compounds in the CO fat samples.

The detection of this compound in the CO oil could be due to fungal contamination on the CO peel. On top of fungal contamination, 10-(β-Dimethyl-aminopropionyl) phenothiazine, which is possibly derived from pesticide, was detected in the CO oleoresin. During the searching and matching of monoisotopic masses of the compounds with the available chemical databases, we found many unmatched compounds. Therefore, these unmatched compounds were named as unknowns. These unknowns had monoisotopic masses ranged between 202.17 and 646.45 Da.

Two unknown compounds were found to be the major compounds in the fat samples—they were relatively abundant in both CO oil and oleoresin. One of the compounds had a molecular formula of C_18_H_34_O_3_, while the molecular formula of another unknown compound was determined. As shown in Table 5, these unknown compounds retained at RT 28.3 min and 35.1 min, respectively. Also, some were the fragment ions of an unknown compound. As this is the first study conducted to identify potential chemical substances and phytochemicals in the CO oil and oleoresin, we merely reported the compounds identified in the CO oil and oleoresin based on the monoisotopic masses obtained from the LC-ESI-MS analysis.

## Conclusion

The LC-ESI-MS analysis gave a full insight into the potent functional compounds and nutritional components in the SCO_2_ extracted CO oil and oleoresin. CO_2_ is the ideal type of organic solvent for the extraction of fat-soluble compounds from plant materials. The CO_2_ extracted CO fat samples had lauric acid, myristic acid, and α-linolenic acid as the major fatty acids in both CO oil and oleoresin. 1-Monolinolein was also the major glyceride in the fat samples; it contains a single fatty acid chain that was covalently bonded through an ester linkage to a glycerol molecule. Two unknown compounds and a peptide (His-Met-Lys) were tentatively identified as the main components of the fat samples. Besides these compounds, phenolics, terpenoids, volatiles, and other aromatic compounds were tentatively identified in both CO oil and oleoresin.

Based on these findings, (E,E)-3,7,11-trimethyl-2,6,10-dodecatrienyl decanoic acid, (+)-3-O-myristoyl-L-1-isovalerin, lacinilene C, hercynine, and 10-(β-dimethyl-aminopropionyl) phenothiazine were the lipids found only in the CO oleoresin. This study has its limitation, where the compounds were only tentatively identified using the LCMS technique. Based on the relative abundance value, a higher level of the long-chain fatty acid such as dihydroxystearic acid was observed in the CO oleoresin than in the oil. Although many compounds are considered as unknown, they have not been identified in the CO fat samples. Future studies may focus on the characterization and identification of these two major unknowns in the CO fat using other analytical technique or a more advanced technology.

## Author Contributions

HK and AA designed this experiment. HK and NA performed the experiments and analyzed the data. HK wrote the manuscript. AA and NA revised and proofread the manuscript.

### Conflict of Interest Statement

The authors declare that the research was conducted in the absence of any commercial or financial relationships that could be construed as a potential conflict of interest.

## References

[B1] Aravindan R. Anbumathi P. Viruthagiri T. (2007). Lipase applications in food industry. Ind. J. Biotechnol. 6, 141–158.

[B2] Askari A. Melville D. B. (1962). The reaction sequence in ergothioneine biosynthesis: hercynine as an intermediate. J. Biol. Chem. 237, 1615–1618. 13862898

[B3] Azlan A. Prasad K. N. Khoo H. E. Abdul-Aziz N. Mohamad A. Ismail A. (2010). Comparison of fatty acids, vitamin E and physicochemical properties of *Canarium odontophyllum* Miq. (dabai), olive and palm oils. J. Food Compos. Anal. 23, 772–776. 10.1016/j.jfca.2010.03.026

[B4] Bagdon R. E. Impellizzeri C. Osadca M. (1962). Studies on the toxicity and metabolism of β-apo-8′-carotenal in dogs. Toxicol. Appl. Pharmacol. 4, 444–456. 10.1016/0041-008X(62)90031-5 13863912

[B5] Basri D. F. Rahman N. S. A. Ali S. S. Zainalabidin S. (2018). The vasorelaxant effect of *Canarium odontophyllum* Miq. (dabai) extract in rat thoracic aorta. J. Basic Appl. Sci. 5, 75–79. 10.1016/j.ejbas.2017.11.004

[B6] Biswas B. Rogers K. McLaughlin F. Daniels D. Yadav A. (2013). Antimicrobial activities of leaf extracts of guava (*Psidium guajava* L.) on two Gram-negative and Gram-positive bacteria. Int. J. Microbiol. 2013:746165. 10.1155/2013/746165 24223039 PMC3817707

[B7] Black H. K. Weedon B. C. L. (1953). Unsaturated fatty acids. Part I. The synthesis of erythrogenic (isanic) and other acetylenic acids. J. Chem. Soc. 40, 1785–1793. 10.1039/jr9530001785

[B8] Carruthers C. (1964). Fatty acid composition of the phosphatide and triglyceride fractions of human epidermis. Proc. Soc. Exp. Biol. Med. 115, 215–218. 10.3181/00379727-115-28873 14117443

[B9] Chew L. Y. Prasad K. N. Amin I. Azrina A. Lau C. Y. (2011). Nutritional composition and antioxidant properties of *Canarium odontophyllum* Miq. (dabai) fruits. J. Food Compos. Anal. 24, 670–677. 10.1016/j.jfca.2011.01.006

[B10] Cho K. H. Hong J. H. Lee K. (2010). Monoacylglycerol (MAG)-oleic acid has stronger antioxidant, anti-atherosclerotic, and protein glycation inhibitory activities than MAG-palmitic acid. J. Med. Food 13, 99–107. 10.1089/jmf.2009.1024 20136442

[B11] D'Evoli L. Lucarini M. Gabrielli P. Aguzzi A. Lombardi-Boccia G. (2015). Nutritional value of Italian pistachios from Bronte (*Pistacia vera*, L.), their nutrients, bioactive compounds and antioxidant activity. Food Nutr. Sci. 6, 1267–1276.

[B12] Díaz-Maroto M. C. Perez-Coello M. S. Cabezudo M. D. (2002). Supercritical carbon dioxide extraction of volatiles from spices: comparison with simultaneous distillation–extraction. J. Chromatogr. A 947, 23–29. 10.1016/S0021-9673(01)01585-0 11873995

[B13] Faridah Hanim S. Azrina A. Khoo H. E. Amin I. (2015). Protective effects of pulp and kernel oils from *Canarium odontophyllum* fruit in normal and hypercholesterolemic rabbits. Int. Food Res. J. 22, 1318–1326.

[B14] Feltes M. M. C. de Oliveira D. Block J. M. Ninow J. L. (2013). The production, benefits, and applications of monoacylglycerols and diacylglycerols of nutritional interest. Food Bioprocess Tech. 6, 17–35. 10.1007/s11947-012-0836-3

[B15] Gross J. Eckhardt G. (1981). Structures of persicaxanthin, persicachrome and other apocarotenols of various fruits. Phytochemistry 20, 2267–2269. 10.1016/0031-9422(81)80127-6

[B16] Güçlü-Üstündag Ö. Temelli F. (2004). Correlating the solubility behavior of minor lipid components in supercritical carbon dioxide. J. Supercrit. Fluids 31, 235–253. 10.1016/j.supflu.2003.12.007

[B17] Ioudina M. Uemura E. (2003). A three amino acid peptide, Gly-Pro-Arg, protects and rescues cell death induced by amyloid β-peptide. Exp. Neurol. 184, 923–929. 10.1016/S0014-4886(03)00314-5 14769384

[B18] IUPAC (1987). Method 2.301: Standard Methods for the Analysis of Oils, Fats and Derivatives, 7th Edition. Durham, NC: International Union of Pure and Applied Chemistry.

[B19] Jelani N. A. A. Azlan A. Ismail A. Khoo H. E. Alinafiah S. M. (2017). Fatty acid profiles and antioxidant properties of dabai oil. ScienceAsia 43, 347–353. 10.2306/scienceasia1513-1874.2017.43.347

[B20] Jürgens U. J. Weckesser J. (1985). Carotenoid-containing outer membrane of *Synechocystis* sp. strain PCC6714. J. Bacteriol. 164, 384–389. 3930470 10.1128/jb.164.1.384-389.1985 PMC214255

[B21] Khoo H. E. (2014). Phenolic Content, Antioxidative Properties, and Cardio-protective Effect of Defatted Dabai Extract. Thesis, Faculty of Medicine and Health Sciences, Universiti Putra Malaysia Available online at: http://psasir.upm.edu.my/51137/

[B22] Khoo H. E. Azlan A. Ismail A. Abas F. (2012). Antioxidative properties of defatted dabai pulp and peel prepared by solid phase extraction. Molecules 17, 9754–9773. 10.3390/molecules17089754 22893021 PMC6268427

[B23] Khoo H. E. Azlan A. Ismail A. Abas F. Hamid M. (2014). Inhibition of oxidative stress and lipid peroxidation by anthocyanins from defatted *Canarium odontophyllum* pericarp and peel using *in vitro* bioassays. PloS ONE 9:e81447. 10.1371/journal.pone.0081447 24416130 PMC3886967

[B24] Lo S.-K. Tan C.-P. Long K. Yussof M. S. A. Lai O.-M. (2008). Diacylglycerol oil—properties, processes and products: a review. Food Bioprocess Tech. 1, 223–233. 10.1007/s11947-007-0049-3

[B25] Lv Z. C. Yin Y. Lin L. J. Peng Y. H. (2014). Chemical constituents from *Canarium pimela* fruits. J. Chin. Med. Mater. 37, 1801–1803. 25895388

[B26] Marina A. M. Man Y. C. Nazimah S. A. H. Amin I. (2009). Chemical properties of virgin coconut oil. J. Am. Oil Chem. Soc. 86, 301–307. 10.1007/s11746-009-1351-1

[B27] McCarty M. F. DiNicolantonio J. J. (2016). Lauric acid-rich medium-chain triglycerides can substitute for other oils in cooking applications and may have limited pathogenicity. Open Heart 3:e000467. 10.1136/openhrt-2016-000467 27547436 PMC4975867

[B28] Momchilova S. Nikolova-Damyanova B. (2003). Stationary phases for silver ion chromatography of lipids: preparation and properties. J. Sep. Sci. 26, 261–270. 10.1002/jssc.200390032

[B29] Mukhopadhyay M. (2009). Extraction and processing with supercritical fluids. J. Chem. Technol. Biotechnol. 84, 6–12. 10.1002/jctb.2072

[B30] Ntumba J. K. Collard L. Taba K. M. Robiette R. (2015). Isolation of a series of fatty acid components of *Ongokea gore* seed (isano) oil and their detailed structural analysis. Lipids 50, 313–322. 10.1007/s11745-014-3984-6 25540043

[B31] Prasad K. N. Chew L. Y. Khoo H. E. Yang B. Azlan A. Ismail A. (2011). Carotenoids and antioxidant capacities from *Canarium odontophyllum* Miq. fruit. Food Chem. 124, 1549–1555. 10.1016/j.foodchem.2010.08.010

[B32] Shakirin F. H. Azlan A. Ismail A. Amom Z. Cheng Yuon L. (2012a). Protective effect of pulp oil extracted from *Canarium odontophyllum* Miq. fruit on blood lipids, lipid peroxidation, and antioxidant status in healthy rabbits. Oxid. Med. Cell. Longev. 2012:840973. 10.1155/2012/840973 22685623 PMC3366250

[B33] Shakirin F. H. Azlan A. Ismail A. Amom Z. Yuon L. C. (2012b). Antiatherosclerotic effect of *Canarium odontophyllum* Miq. fruit parts in rabbits fed high cholesterol diet. Evid. Based Complement. Alternat. Med. 2012:838604. 10.1155/2012/838604 22811751 PMC3395265

[B34] Siri-Tarino P. W. Sun Q. Hu F. B. Krauss R. M. (2010). Saturated fatty acids and risk of coronary heart disease: modulation by replacement nutrients. Curr. Atheroscler. Rep. 12, 384–390. 10.1007/s11883-010-0131-6 20711693 PMC2943062

[B35] Song C. (2015). Biosynthesis of Acylphloroglucinol Glucosides in Strawberry Fruit. Thesis, Technische Universität München. Available online at: https://mediatum.ub.tum.de/1271772

[B36] Spiteller G. (1998). Linoleic acid peroxidation—the dominant lipid peroxidation process in low density lipoprotein—and its relationship to chronic diseases. Chem. Phys. Lipids 95, 105–162. 10.1016/S0009-3084(98)00091-7 9853364

[B37] Zock P. L. de Vries J. H. Katan M. B. (1994). Impact of myristic acid versus palmitic acid on serum lipid and lipoprotein levels in healthy women and men. Arterioscler. Thromb. 14, 567–575. 8148355 10.1161/01.atv.14.4.567

